# Effects of *In Vitro* Fermented *Pleurotus eryngii* on Intestinal Barrier Integrity and Immunomodulation in a Lipopolysaccharide-Induced Colonic Model

**DOI:** 10.3390/biomedicines13020430

**Published:** 2025-02-11

**Authors:** Evangelia N. Kerezoudi, Georgia Saxami, Georgios I. Zervakis, Vasiliki Pletsa, Robert J. Brummer, Adamantini Kyriacou, Ignacio Rangel

**Affiliations:** 1 Nutrition-Gut-Brain Interactions Research Centre, School of Medical Sciences, Örebro University, 70182 Örebro, Sweden; robert.brummer@oru.se (R.J.B.); ignacio.rangel@oru.se (I.R.); 2 Department of Nutrition and Dietetics, Harokopio University, 17676 Athens, Greece; saxamigeorgia@hotmail.com (G.S.); kyriacou@hua.gr (A.K.); 3 Institute of Technology of Agricultural Products, Hellenic Agricultural Organization—DIMITRA (ELGO-DIMITRA), Sof. Venizelou 1, 14123 Athens, Greece; 4 Laboratory of General and Agricultural Microbiology, Department of Crop Science, Agricultural University of Athens, 11855 Athens, Greece; zervakis@aua.gr; 5 National Hellenic Research Foundation, Institute of Chemical Biology, 11635 Athens, Greece; vpletsa@eie.gr

**Keywords:** *Pleurotus eryngii*, lipopolysaccharides, gut barrier function, tight junctions, immune response

## Abstract

**Background**: This study investigates the impact of fermentation supernatants (FSs) from *Pleurotus eryngii* whole mushrooms (PEWS), as well as its subcomponents, digested (PEWSD) and extracted (PEWSE) forms, on intestinal barrier function and immune modulation in lipopolysaccharide (LPS) -stimulated Caco-2 cells. **Methods**: Gene expression of tight junction (TJs) genes, cytokines, and key immune/metabolic receptors was assessed via qRT-PCR, while cytokine protein levels were measured using ELISA to explore post-transcriptional regulation. **Results**: LPS challenge significantly downregulated TJs *zonula occludens*-1 (*ZO-1*,) *occludin*, and *claudin-1*, compromising epithelial integrity. Treatment with FS-PEWS notably restored *ZO-1* and *occludin* expression, outperforming FS-PEWSD and FS-PEWSE, which only partially mitigated the LPS-induced damage. FS-PEWS further demonstrated potent immunomodulatory effects, upregulating anti-inflammatory *IL-10* and pro-inflammatory cytokines such as *IL-8* and *TNF-α*. The activation of key receptors like *TLR-2* and *mTOR* suggests that FS-PEWS modulates critical immune and metabolic pathways, such as *NF-kB* signaling, to maintain immune homeostasis. Although mRNA expression of pro-inflammatory cytokines was altered, no corresponding protein release was detected, suggesting potential post-transcriptional regulation. **Conclusions**: FS-PEWS preserves intestinal barrier integrity and modulates immune responses, particularly in low-grade inflammation, highlighting the whole food matrix’s role in enhancing its bioactivity and functional food potential.

## 1. Introduction

The intestinal barrier represents a pivotal physical and immunological partition that meticulously segregates the host’s mucosal environment from the luminal contents within the gastrointestinal tract. This barrier operates as a selectively permeable membrane, essential for the absorption of vital nutrients and water, while robustly defending against the ingress of harmful agents, including antigens and microorganisms, into the submucosal tissues. Integral to its function are specialized intercellular junctional complexes—desmosomes, adherens junctions, and tight junctions (TJs)—which intricately regulate paracellular spaces between epithelial cells, thus preserving homeostasis [1,2]. Compromise of the TJs leads to intestinal wall leakage syndrome, characterized by heightened intestinal permeability, a phenomenon closely associated with a broad range of disorders, encompassing inflammatory and autoimmune conditions as well as metabolic and neurological diseases [3].

Another important component of the intestinal barrier, the gut microbiota (GM), comprises approximately 10^13^ bacterial cells, comprising a complex ecosystem of more than 250 species of bacteria, fungi, protozoa, and viruses, all existing in symbiosis with the host organism [4]. This dynamic system inhabiting especially the colonic segment of the gastrointestinal tract evolves throughout an individual’s lifetime and plays a pivotal role in maintaining overall health through its intricate interplay with dietary components and host immune responses [5]. Aging is intricately linked with substantial shifts in gut microbiota composition and a decline in barrier function, leading to dysbiosis and increased intestinal permeability. Studies consistently highlight a higher prevalence of aberration of intestinal permeability in older adults, contributing significantly to systemic inflammation and the onset of age-related diseases [6].

This age-related decline in gut barrier function is compounded by “microbaging” and increased susceptibility to immunosenescence, which is characterized by a shift from an anti-inflammatory to a pro-inflammatory cytokine profile, a phenomenon referred to as “inflammaging” [7]. Recent studies underscore the pivotal roles of cytokines in modulating gut barrier integrity and immune responses. For instance, Thevaranjan et al. showed that with the onset of age-associated intestinal barrier dysfunction, microbial products infiltrated the bloodstream of aged mice, triggering systemic inflammation [8]. This was evidenced by elevated serum levels of interleukin IL-6, among other inflammatory indicators [8]. Numerous studies have consistently demonstrated the involvement of elevated concentrations of pro-inflammatory cytokines, such as tumor necrosis factor-alpha (TNF-α) and IL-1β, in downregulating the expression of tight junction proteins, including zonulin and claudins. This dysregulation contributes to enhanced epithelial barrier permeability, which may perpetuate the inflammatory cascade [9].

Lipopolysaccharide (LPS), a component of the outer membrane of Gram-negative bacteria, is regarded as a highly potent inducer of gut inflammation. It can trigger various rapid stress responses through Toll-like receptor (TLR)-mediated signaling pathways in intestinal epithelial cells. It enhances the production of inflammatory mediators, such as cytokines, by activating inflammatory signaling cascades like the NF-kB pathway. Additionally, it causes disruption of TJs and is also involved in the regulation of autophagy by activating the mechanistic target of rapamycin (mTOR) signaling pathway [10,11].

The interaction between diet, the gut microbiota, and host health has led to significant interest in prebiotics, defined as “non-digestible substrates that are selectively utilised by host microorganisms conferring a health benefit” [12], in modulating gastrointestinal function and enhancing barrier integrity [13]. Prebiotics exert profound effects on gut health by influencing metabolic processes and bolstering immune defences, underscoring their therapeutic potential in managing gut-related disorders [14]. Mushrooms, such as those produced by species the genus *Pleurotus* (Basidiomycota, Agaricales) and notably by *P. eryngii* (“King Oyster mushroom”), are esteemed for their rich bioactive profiles, prominently featuring *β*-glucans among other compounds. Research has underscored their ability to mitigate ’leaky gut’ by enhancing mucosal immunity and modulating inflammatory pathways critical for gut health maintenance [15].

*In vitro* studies have shown that the fermented *P. eryngii* whole food matrix can protect an LPS-compromised intestinal barrier by upregulating *TJ* gene expression [16]. It has also been found to influence immunomodulation in human macrophages and peripheral blood mononuclear cells [17]. Nevertheless, a critical gap remains in understanding its effects on intestinal immunoregulation and molecular signaling pathways, particularly under conditions of low-grade inflammation. Addressing this gap necessitates a comparative analysis of *P. eryngii* in distinct forms: the whole fruit mushroom body, its *in vitro*-digested form, which simulates gastrointestinal processing, and its *β*-glucan-enriched extract, isolating a key bioactive component for targeted exploration.

To contextualize these substrates for aging populations, fecal inocula from healthy individuals over 65 years old were used for fermentation, mirroring the gut microbiota composition typical of this demographic. Their effects were evaluated in a Caco-2 LPS-stimulated colonic model, previously developed [16] but applied here for the first time to assess the impact of different *P. eryngii* substrates (PEWS, PEWSD, PEWSE) on immune responses and signaling cascades. This innovative framework captures the physiological nuances of aging, a context marked by compromised intestinal barrier function and chronic, low-grade inflammation.

Thus, the objective of this study was to systematically assess the effects of *P. eryngii* mushroom-based substrates on intestinal barrier integrity, immunomodulatory potential, and signaling pathways. By unveiling mechanistic insights into how these substrates modulate cytokine signaling and tight junction dynamics, this research aims to improve the understanding of *P. eryngii* bioactivities and to explore its potential as a functional food ingredient for addressing age-related intestinal and immune dysfunction.

## 2. Materials and Methods

### 2.1. Generation of Fermentation-Derived Supernatants

Fermentation supernatants (FSs) were produced via *in vitro* static batch culture fermentation over a 24 h period, utilizing fecal samples from five healthy older adult donors aged 65 years or older, in accordance with a previously described methodology [18]. The fermentation was carried out using the microbiota present in the fecal samples, without the addition of specific bacterial strains. The substrates employed comprised 2% (*w*/*v*) lyophilized powder from the whole fruit body (mushroom) of *P. eryngii* strain LGAM 216 (PEWS). The mushrooms were subjected to lyophilization for 24 h using a Telstar Cryodos system and subsequently processed into a fine powder through milling. Total and *α*-glucan levels were quantified using an assay kit from Megazyme Ltd. (Wicklow, Ireland), while the *β*-glucan content was determined by subtracting the measured *α*-glucan fraction from the total glucan concentration. The *β*-glucan composition, reported as a percentage of dry weight (% *w*/*w*), varied across the preparations: the intact fruiting body (PEWS) contained 38.7 ± 5.4%, the enzymatically digested preparation (1% *w*/*v*, PEWSD) yielded 39.8 ± 4.8%, and the hot water extract (1% *w*/*v*, PEWSE) exhibited the highest β-glucan content at 49.7 ± 4.7%. A negative control (NC) was also included, consisting of the same fermentation basal medium, as previously outlined [19], devoid of any carbon source. Post-fermentation, the samples were centrifuged at 10,000× *g* for 30 min at 4 °C to isolate the supernatant. These supernatants were then filtered through a 0.22 µm pore filter (Millex^®^, Merck KGaA, Darmstadt, Germany) and stored at −20 °C.

### 2.2. Cell Culture of Caco-2 Cells and Bacterial LPS

The Caco-2 human colon adenocarcinoma cell line was procured from the American Type Culture Collection (ATCC). Cultivation was conducted in Dulbecco’s Modified Eagle Medium (DMEM) containing stable glutamine (Biosera, Nuaille, France), supplemented with 1% penicillin/streptomycin (10,000 U/mL, Biochrom AG, Berlin, Germany) and 10% (*v*/*v*) fetal bovine serum (Biochrom AG). The cells were maintained at 37 °C in a humidified environment with 5% CO_2_. Experiments utilized cells from passages 20 to 30. Bacterial lipopolysaccharides from *Escherichia coli* O55 (Cayman Chemical Company, Ann Arbor, MI, USA) were employed to provoke a disruption in intestinal barrier permeability.

### 2.3. Exposure of Caco-2 Cells to FSs and LPS Treatment

Caco-2 cells were initially seeded in 6-well plates at a density of 130,000 cells per well, utilizing DMEM complete medium. Subsequently, to evaluate the protective effects of the FSs on intestinal barrier permeability, the cells were treated under conditions where they were pre-exposed to the FSs before the addition of LPS. This process involved subjecting Caco-2 cells to the FSs from various forms of the mushrooms or to negative controls for 48 h at a final concentration of 2% *v*/*v*, followed by exposure to LPS (100 ng/mL) for 24 h at 37 °C in a 5% CO_2_ environment, as defined by our prior standardization data [16]. In addition, cells treated only with LPS (cells + LPS) were used as an internal control to assess the effect of LPS on the impairment of the intestinal barrier.

### 2.4. Preparation of Samples for TJ Gene Expression

RNA extraction was conducted using NucleoZOL reagent (MACHEREY-NAGEL GmbH & Co. KG, Dueren, Germany), following the protocol provided by the manufacturer. The integrity and concentration of the isolated RNA were assessed via spectrophotometric analysis. Subsequently, 1 ug of total RNA was reverse-transcribed into complementary DNA (cDNA) using the PrimeScript First Strand cDNA Synthesis Kit (Takara Bio Inc., Kusatsu, Shiga, Japan). Quantitative real-time PCR was conducted using the StepOne PCR System and MicroAmp^®^ Fast Optical 96-Well Reaction Plates (Thermo Fisher Scientific, Waltham, MA, USA). The KAPA SYBR^®^ FAST qPCR Kit (Kapa Biosystems, Wilmington, MA, USA) was utilized, following the recommended protocol: an initial phase at 95 °C for 3 min, followed by 40 cycles of 95 °C for 15 s and 60 °C for 1 min. The housekeeping gene *b-actin* was used as an internal control, with untreated cells serving as the reference sample. Each reaction was performed in duplicate (100 ng/ul of cDNA). The primer sequences (200 nm) for *TJs* genes (*ZO-1*, *occludin,* and *claudin-1*) are presented in Table 1. RT-PCR was conducted using the ABI StepOne system (Applied Biosystems, Waltham, MA, USA). Primer specificity was confirmed through melting curve analysis. For the relative quantification of the transcripts, the formula RQ = 2^−∆∆Ct^ was applied.

### 2.5. Preparation of Samples for Cytokine and Receptor Gene Expression

RNA was extracted and its quality and quantity were assessed as previously described. Using the High-Capacity cDNA Reverse Transcription Kit (Applied Biosystems, Waltham, MA, USA), 1 ug of total RNA was transcribed into cDNA. Quantitative real-time PCR (qRT-PCR) was conducted with the SensiMix™ SYBR^®^ Low-ROX Kit (Nordic Biosite AB, Täby, Sweden) in 96-Well PCR Reaction Plates with full skirt (Sarstedt, Inc., Hildesheim, Germany). The protocol entailed an initial denaturation at 95 °C for 10 min, followed by 40 cycles at 95 °C for 15 s and 60 °C for 15 s. *β*-actin served as the housekeeping gene and untreated cells were used as the reference. Reactions were performed in duplicate (100 ng/ul of cDNA) using primers (250 nM) for cytokine (*IL-1β*, *TNF-α*, *IFN-γ*, *IL-6*, *IL-8*, *IL-10*, *IL-13*, *IL-17*) and receptor (*dectin-1*, *TLR-2*, *TLR-4*, *NF-kB*, *complement receptor-3* (*CR3*), *mTOR*) genes, as listed in Table 1. RT-PCR was conducted using the BioRad CFX96 Real-Time System C1600 Touch Thermal Cycler and data visualization was performed using Bio-Rad CFX Manager version 3.1, 2012 (BioRad Laboratories, Berkeley, CA, USA). Primer specificity was verified through melting curve analysis and the relative quantification of transcripts was determined using the 2^−ΔΔCt^ method.

### 2.6. Quantification of Cytokines’ Release

Selected secreted IFN-γ, IL-1β, IL-6, IL-10, and TNF-α cytokines were measured in the Caco-2 cell supernatant obtained after centrifugation at 10, 000× *g* for 5 min at 4 °C using the MSD V-Plex Panel (Meso Scale Discovery, Rockville, MD, USA), according to the manufacturer’s instructions. Diluted supernatants (dilution 1:1 up to 1:10) were analyzed in duplicate and the detection range of the assay is shown in Table S1.

### 2.7. Statistical Analysis

The data, i.e., TJ, cytokine, and receptor genes, were calculated by taking into consideration all of the technical replicates of all biological replicates, referring to each gene of interest and normalizing them to the reference gene of each technical run. Normality of distribution of continuous variables was tested by the Shapiro–Wilk test and by visualizing the data in histograms. Continuous variables were characterized by their median values and interquartile ranges [(IQR, (Q1, Q3)]. For all statistical analyses and comparative evaluations between the controls and the different treatment groups, non-parametric methods were utilized, specifically employing the Wilcoxon signed-rank test. Statistical analyses were executed using IBM^®^ SPSS^®^ Statistics version 28, with the significance level set at 5% (*p* < 0.05).

## 3. Results

### 3.1. Preservative Influence of P. eryngii FSs on Intestinal Barrier’s Integrity via TJ Gene Upregulation

**Figure 1:**
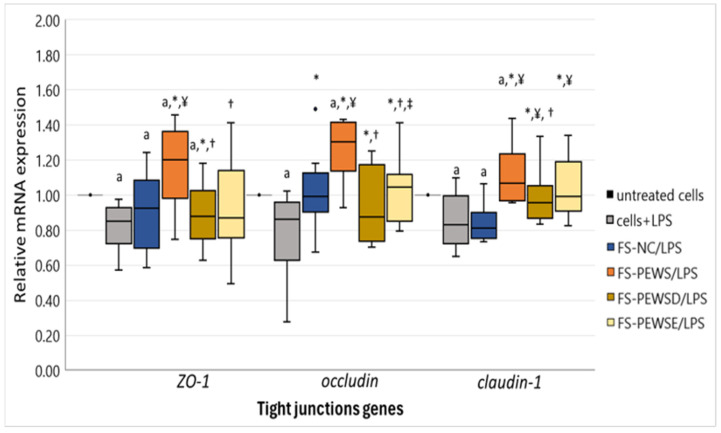
The relative expression of TJ genes in response to incubation (2% *v*/*v* for 48 h) with FS-NC, FS-PEWS, FS-PEWSD and FS-PEWSE from a total of five fecal donors in the LPS-stimulated (100 ng/mL for 24 h) Caco-2 cells. Data are expressed as mRNA expression (normalized to that of *β*-actin) relative to untreated cells as the mean ± SD of two independent experiments. Untreated cells: culture cells without any effect, Cells+LPS: culture cells stimulated only with LPS, FS-NC: FSs of the negative control (basal medium with no carbohydrate source); FS-PEWS: *P. eryngii* lyophilized powder from the whole fruit body (mushroom); FS-PEWSD: enzyme-digested PEWS; FS-PEWSE: hot-water extract of PEWS; a: statistically significant compared to untreated cells; *: statistically significant compared to LPS; ¥: statistically significant compared to FS-NC/LPS; †: statistically significant compared to FS-PEWS/LPS; ‡: statistically significant compared to FS-PEWSD/LPS; *p* < 0.05 (Wilcoxon signed-rank test).

The prospective impacts of the various mushroom-derived fermented substances on the integrity of the intestinal epithelial barrier within LPS-stimulated Caco-2 cell monolayers were assessed by quantifying the expression levels of *TJ* genes pivotal to barrier formation, functionality, and overall integrity. Figure 1 delineates the expression profiles of the *TJ* genes subsequent to pre-incubation of Caco-2 cells with the FSs sourced from the total of all five elderly individuals.

LPS exposure of Caco-2 cells led to a significant downregulation of all *TJ* genes relative to untreated cells (*p* < 0.001). Incubation with FS-NC elevated *occludin* gene expression [0.95 (0.84, 1.10)] in comparison to solely LPS-treated cells [0.87 (0.65, 0.96)] (*p* = 0.033). However, this treatment did not elicit a similar effect on *ZO-1* [0.92 (0.64, 0.98)] and *claudin-1* [0.83 (0.71, 0.94)] expression levels, which were also significantly reduced compared to the untreated control cells (p_zo-1_ = 0.008, p_occludin_ < 0.001).

All distinct mushroom FSs effectively counteracted the LPS-induced suppression of *TJ* gene expression, except for FS-PEWSE, which, despite elevating *ZO-1* gene expression, failed to fully mitigate the LPS-induced effect.

The FSs of the whole food matrix of *P. eryngii* mushrooms demonstrated superior efficacy across all examined substrates—untreated cells, LPS-stimulated cells, FS-NC, FS-PEWSD, and FS-PEWSE—in *TJ* gene expression (*p* < 0.05) (Table S2). The sole exception was *claudin-1*, where FS-PEWS and FS-PEWSE exhibited comparably elevated expression levels (*p* = 0.321).

The FSs of the digested *P. eryngii* significantly restored the expression levels of *occludin* [0.88 (0.71, 1.16)] and *claudin-1* [0.96 (0.86, 1.08)] genes, similar to those observed in untreated cells. However, this treatment resulted in a substantial reduction in the *ZO-1* [0.88 (0.76, 1.03)] expression level compared to untreated controls (*p* = 0.034). Additionally, the effects on *ZO-1* (*p* = 0.814) and *occludin* (*p* = 0.526) expression levels were akin to those observed with FS-NC, while FS-PEWSD significantly enhanced *claudin-1* expression compared to the negative control (*p* = 0.004).

Finally, FS-PEWSE exhibited effects analogous to those of FS-PEWSD, except for a substantially higher *occludin* expression level detected after its treatment [FS-PEWSD: 0.88 (0.71, 1.16), FS-PEWSE: 1.04 (0.87, 1.10) (*p* = 0.043)]. Incubation with the FSs of the purified extracted *P. eryngii* successfully preserved the integrity of the intestinal barrier against LPS-induced stress, normalizing the expression levels of all *TJ* genes to those seen in untreated cells (*p* > 0.05). Markedly, *claudin-1* [1.06 (0.88, 1.23)] expression was significantly higher compared to the negative control [(0.83 (0.71, 0.94)] (*p* < 0.001).

In summary, all tested *P. eryngii* substrates displayed a positive impact on the preservation of the intestinal barrier by promoting uniform expression or overexpression of *TJ* genes associated with intestinal paracellular permeability. However, the most pronounced effect was observed with FS-PEWS, which exhibited the highest and statistically significant increases in gene expression compared to all other substrates tested, especially for *ZO-1* and *occludin*.

### 3.2. Immunomodulatory Impact of FS-PEWS on Cytokine mRNA Expression Levels in the LPS-Stimulated Caco-2 Cells

**Figure 2:**
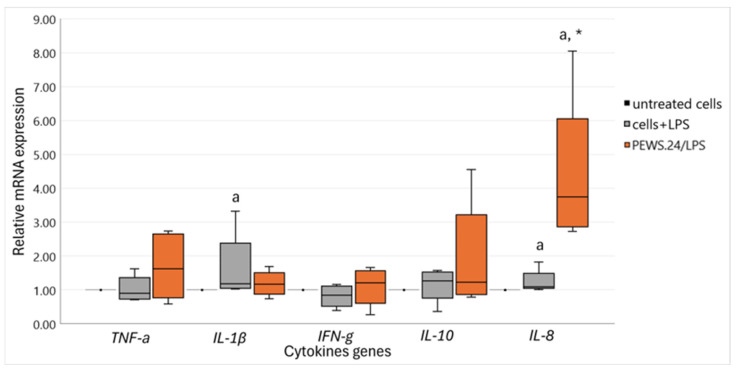
The relative expression of cytokine genes in response to incubation (2% *v*/*v* for 48 h) with FS-PEWS from a total of five fecal donors in the LPS-stimulated (100 ng/ mL for 24 h) Caco-2 cells. Data are expressed as mRNA expression (normalized to that of *β*-actin) relative to untreated cells as the mean ± SD of two independent experiments. Untreated cells: culture cells without any effect, Cells + LPS: culture cells stimulated only with LPS, FS-PEWS: *P. eryngii* lyophilized powder from the whole fruit body (mushroom); a: statistically significant compared to untreated cells; *: statistically significant compared to LPS; *p* < 0.05 (Wilcoxon signed-rank test).

Taking into consideration the results of FS-PEWS’ effect on the protection of intestinal barrier integrity, a subsequent targeted analysis focusing solely on this specific substrate’s intestinal immunomodulatory potential was performed. Figure 2 demonstrates the results regarding the cytokine expression levels, including the pro-inflammatory cytokines *IL-1β*, *IL-8*, *TNF-a*, and *IFN-γ* as well as the anti-inflammatory cytokine *IL-10*, following exposure to FS-PEWS in the LPS-stimulated Caco-2 cell line. *IL-6*, *IL-13*, and *IL-17* gene expression levels were also tested, but their levels were below the detection limit; therefore, these results are not included in the analysis.

Caco-2 cells treated only with LPS exhibited notable increases in the expression levels of *IL-1β* [1.18 (1.06, 2.39)] and *IL-8* [1.09 (1.05, 1.49)] pro-inflammatory cytokines, which demonstrated statistical significance compared to untreated cells (p_IL-1β_ = 0.043, p_IL-8_ = 0.042). LPS stimulation for 24 h at the tested concentration did not seem to influence the rest of the pro-inflammatory cytokine expression levels, i.e., *TNF-α* [0.90 (0.74, 1.36)] or *IFN-γ* [0.84 (0.53, 1.12)], since their levels were even or minimally diminished in relation to untreated controls, correspondingly (p_TNF-a_ = 0.786, p_IFN-γ_ = 0.279). On the contrary, a minor (not significant *p* = 0.500) increase in the expression level of *IL-10* [1.27 (0.76, 1.54)] anti-inflammatory cytokine was observed in comparison to the untreated cells.

When Caco-2 cells were incubated with the FSs of the *P. eryngii* whole food matrix, all cytokine levels, both pro- and anti- inflammatory, increased, followed by large inter-individual variation effects. Elevated levels of *IL-10* [1.23 (0.87,3.22)], *TNF-α* [1.63 (0.77, 2.66)], and *IL-8* [3.75 (2.87, 6.05)] were observed, where especially in the case of *IL-8* a noteworthy increase compared to both untreated and LPS-stressed cells was detected (p_IL-8_ = 0.043).

### 3.3. Effect of FS-PEWS in the LPS-Stimulated Caco-2 Cells Linked to Intestinal Immune Regulation and Metabolism-Related Receptor mRNA Expression Levels

**Figure 3:**
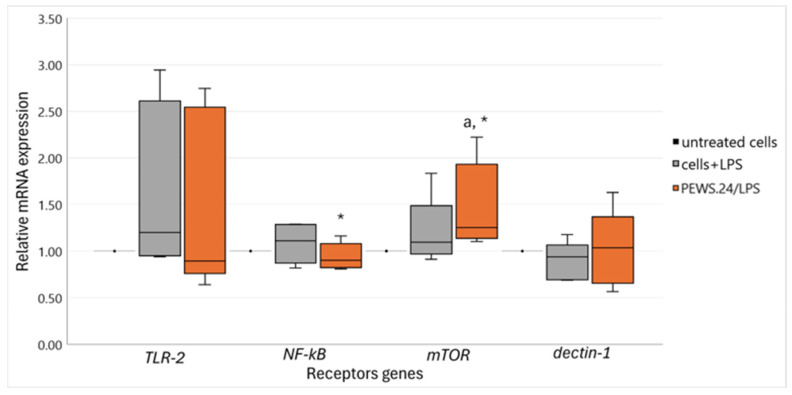
The relative expression of receptor genes in response to incubation (2% *v*/*v* for 48 h) with FS-PEWS from a total of five fecal donors in the LPS-stimulated (100 ng/ mL for 24 h) Caco-2 cells. Data are expressed as mRNA expression (normalized to that of β-actin) relative to untreated cells as the mean ± SD of two independent experiments. Untreated cells: culture cells without any effect, Cells + LPS: culture cells stimulated only with LPS, FS-PEWS: *P. eryngii* lyophilized powder from the whole fruit body (mushroom); a: statistically significant compared to untreated cells; *: statistically significant compared to LPS; *p* < 0.05 (Wilcoxon signed-rank test).

To elucidate how FS-PEWS safeguards gut permeability under LPS-induced stress while modulating intestinal immune responses via multiple epithelial receptors, we analyzed the expression levels of specific receptors. Figure 3 presents data on *TLR-2*, *NF-kB*, *mTOR*, and *dectin-1* gene expression levels, as *TLR-4* and *CR3* remained undetectable.

LPS stimulation of Caco-2 cells resulted in upregulated expression levels of *TLR-2* [1.20 (0.95, 2.61)], *NF-kB* [1.11 (0.87, 2.61)], and *mTOR* [1.10 (0.97, 1.49)] and attenuated *dectin-1* [0.94 (0.70, 1.07)] levels, although these changes were not statistically significant compared to untreated cells (*p* > 0.05). By contrast, treatment with FS-PEWS did not alter the expression of *TLR-2* [0.89 (0.76, 2.55)], but it significantly amplified the expression of *mTOR* [1.25 (1.14, 1.93)], showing an increase compared to both untreated and LPS-stressed cells (*p* = 0.043). FS-PEWS treatment restored *NF-kB* [1.25 (1.14, 1.93)] expression to the level seen in untreated cells while significantly reducing it compared to that in LPS-stimulated cells (*p* = 0.043). No significant differences were observed in *dectin-1* expression across the treatments [LPS: 0.94 (0.70, 1.07), FS-PEWS: 1.04 (0.65, 1.37)] (*p* > 0.05).

### 3.4. Undetectable Cytokines Released in Caco-2 Cell Culture Supernatants

A high sensitivity V-PLEX analysis was conducted to correlate the gene expression results with protein release. The protein secretion of specific cytokines associated with intestinal inflammation was assessed in the cell culture supernatants of Caco-2 cells. Neither FS-PEWS mushroom treatment nor stimulation with LPS alone as a stressor showed any detectable effect on the release of IFN-γ, IL-1β, IL-6, IL-10, and TNF-α.

## 4. Discussion

This study examined the *in vitro* impact of fecal fermentation supernatants derived from *P. eryngii* mushrooms on tight junction gene expression and immunomodulatory activity in Caco-2 intestinal epithelial cells subjected to bacterial lipopolysaccharide. The *P. eryngii* FSs of healthy elderly individuals, analyzed in their whole food matrix, *in vitro*-digested, and extracted forms, demonstrated significant potential in enhancing gut barrier integrity and modulating inflammatory activity with subsequent immune signaling pathway involvement.

Despite the small sample size, we used fecal donors from healthy elderly individuals, ensuring that the observed effects would not be confounded by underlying pathologies, thereby providing a clearer understanding of the FSs’ potential in maintaining gut health and regulating immune responses. In this regard, our data supports that FS-PEWS treatment remarkably mitigated the LPS-induced suppression of *TJ* gene expression, particularly *ZO-1* and *occludin*, hence protecting intestinal barrier integrity, which is in line with the results of our previous *in vitro* study [16]. FS-PEWSD and FS-PEWSE also demonstrated beneficial effects on *TJ* gene expression, albeit to a lesser extent than FS-PEWS. FS-PEWSD significantly enhanced *claudin-1* expression, while FS-PEWSE showed a more selective effect, being effective only for *occludin* and *claudin-1* in maintaining barrier integrity. These observations are in line with the evidence linked to these FSs’ prebiotic potential, as described previously [18], suggesting that the whole food matrix of *P. eryngii* is superior in promoting gut barrier function compared to its digested or highly enriched with *β*-glucans extracted form, which was the reason that the further analysis focused exclusively to FS-PEWS.

Pro-inflammatory cytokines such as IL-1β, IL-8, TNF-α, and IFN-γ are implicated in inducing inflammation and disrupting tight junctions, thereby compromising barrier function. Conversely, anti-inflammatory cytokines like IL-10 play crucial roles in attenuating inflammatory responses and maintaining barrier integrity, highlighting their therapeutic potential in mitigating gut permeability issues associated with aging [27,28]. To elucidate how FS-PEWS safeguards gut permeability under LPS-induced stress, we analyzed the expression levels of key immune and metabolic receptors. Our results confirmed that LPS in significantly lower dosage (100 ng/mL) compared to the ones broadly used in the already existing literature simulating endotoxemia conditions (4–50 ug/mL) [29,30] can lead to low-level intestinal inflammation, as evidenced by the substantial downregulation of all *TJ* genes and the upregulation of pro-inflammatory cytokines *IL-1β* and *IL-8*, serving as a robust platform for evaluating therapeutic interventions.

Building upon these findings, FS-PEWS exhibited significant immunomodulatory effects by elevating the expression levels of the anti-inflammatory cytokine *IL-10* and pro-inflammatory cytokines *TNF-α* and *IL-8*. Similar pro-inflammatory effects have been shown from the stimulation of THP-1 macrophages with *β*-1,6-glucan from *Pleurotus citrinopileatus* mushroom extract (PCPS) or *β*-glucans from *Shiitake* mushrooms, leading to the overexpression of *TNF-α* and *IL-1β*, and even *IL-8*, *NF-kB*, and *IL-10*, respectively [31,32]. Analogous effects were presented by Case et al., where monocytes isolated from human peripheral blood mononuclear cells (PBMCs) exhibited increased secretion of TNF and IL-6 after exposure to whole mushroom powder from *Agaricus bisporus*. The authors proposed that this immune response was attributed to the low-molecular-weight *β*-glucan content of the mushroom [33]. This ability prompts a nuanced discussion on the balance between pro-inflammatory and anti-inflammatory cytokines. Traditionally, pro-inflammatory cytokines like TNF-α and IL-8 initiate and propagate immune responses, while IL-10 acts to dampen inflammation and promotes tissue repair. However, a balanced immune response, where the levels of both pro-inflammatory and anti-inflammatory cytokines are elevated in a controlled manner, may offer strategic benefits. Specifically, a mild inflammatory response can help to maintain immune system homeostasis by ensuring a balanced activation of immune pathways [34]. Such balance is crucial for effective pathogen clearance, immune cell recruitment, and tissue homeostasis maintenance, which may prove beneficial in managing conditions marked by dysregulated immune responses, where excessive inflammation is implicated. It acts as a “training” mechanism for the immune system, helping it to differentiate between harmful and non-harmful stimuli and to mount appropriate responses. In such scenarios, interventions that promote a balanced cytokine profile, akin to that potentially induced by FS-PEWS, may offer therapeutic benefits by attenuating inflammation while preserving immune responsiveness.

The LPS-stressed Caco-2 experimental design used in this study represents an intestinal low-grade inflammation model. These types of *in vitro* models could act as valuable tools for assessing the protective effects of molecules in the early stages of gut barrier dysfunction, even in phenotypically healthy individuals with potential underlying low-grade inflammation. Even under *in vivo* conditions, Stehle et al. showed, using a cross-sectional comparison, that in apparently healthy adults over the age of 60, dysfunction of the intestinal barrier, as indicated by elevated levels of microbial translocation markers such as LPS-binding protein (LBP) in blood plasma, was linked to impaired physical function and increased inflammation, e.g., IL-6 and TNF-α [35]. So, LPS-induced stress models mimic conditions where subtle changes in cytokine profiles and immune cell responses contribute to initial disruptions in barrier function, making them ideal for studying interventions aimed at preserving gut integrity before clinical manifestations occur.

Despite the changes in cytokine mRNA levels, the V-PLEX analysis revealed no detectable protein release of IFN-γ, IL-1β, IL-6, IL-10, and TNF-α in the supernatants of Caco-2 cells following FS-PEWS treatment or LPS stimulation alone. To our knowledge, no other study has assessed the effect on immunomodulation of these specific LPS stimulation conditions in Caco-2 cells. However, other studies have detected the mRNA expression and protein release of proinflammatory cytokines, e.g., TNF-α, IL-1β, and IL-6, after LPS (10 ug/mL for 12 h) stimulation of other colonic cell lines, i.e., rat IEC-6 cell line [36]. This discrepancy between mRNA expression and protein release could be attributed to the time-dependent nature of cytokine secretion, where mRNA expression might peak earlier than protein synthesis and release, which could occur outside our experimental timeframe [37]. Also, discrepancies could be related to post-transcriptional and post-translational regulatory mechanisms that control cytokine secretion depending on the conditions of the methodological model used [38,39].

Drawing from this evidence and in our pursuit to further investigate the connection between the enhancement of the intestinal barrier by FS-PEWS and its immunomodulatory effects, we evaluated the regulation of receptor genes involved in immune activation. Our results revealed that while significant changes were observed in *IL-8* and *IL-1b* after cell stimulation with LPS alone, other cytokines and receptors such as *TNF-α*, *NF-kB*, *IFN-γ*, *IL-10*, *TLR-2*, and *mTOR* did not show statistically significant differences compared to the untreated cells. On a similar line, FS-PEWS treatment significantly upregulated *TLR-2* and *mTOR* expression, suggesting the activation of pivotal signaling cascades involved in immune surveillance and metabolic regulation, like PI3K/Akt/mTOR and MyD88-dependent NF-kB signaling, which regulate immune responses, inflammation, and cellular metabolism essential for intestinal epithelial function [40]. Additionally, FS-PEWS normalized *NF-kB* expression to the level seen in untreated cells, indicating its role in mitigating inflammatory signaling pathways. The significant modulation of *IL-8* and *NF-kB* pathways, along with the enhancement of *TJ* genes, supports this hypothesis. Given that FS-PEWS are rich in *β*-glucans, and these molecules are known for their immunomodulatory properties, it is plausible to speculate that *β*-glucans are responsible for the observed effects. These polysaccharides interact with a variety of immunological receptors, such as dectin-1, CR3, and TLR2/6, thereby contributing to their immunological efficacy [41].

However, the lack of significant change in *dectin-1* expression and the inability to detect *CR3* suggest that *β*-glucans might not exert their effects through these receptors alone, especially in intestinal epithelial cells. *Dectin-1* and *CR3* are extensively expressed within the myeloid cell lineage, encompassing macrophages, monocytes, dendritic cells (DCs), neutrophils, and large granular lymphocytes [42,43]. On the one hand, the literature presents conflicting evidence regarding the presence of dectin-1 in intestinal epithelial cells. Specifically, Volman et al. showed that human enterocytes isolated from ileum or colon biopsies and the Caco-2 NF-kB reporter enterocyte cell line did not seem to express functional dectin-1 on cells’ extracellular membrane [44]. On the contrary, Cohen-Kedar et al. referred to the potential of fungal or yeast *β*-glucans to activate dectin-1 receptor in primary human intestinal epithelial cells as well as in HT-29 and SW480 cell lines [45]. While previous research has documented the expression of *CR3* in Caco-2 cell models [46], we did not detect *CR3* expression in untreated Caco-2 cells or after LPS stimulation. For instance, Satyam et al. observed an elevation in *CR3* mRNA levels following 24 h stimulation with LPS at a concentration of 100 ng/mL in a Caco-2 model [47]. Hence, the outcomes of this study diverge partly from the existing literature since our results indicate that neither dectin-1 nor CR3 is functionally active in Caco-2 cells. Therefore, the immunomodulatory effects of FS-PEWS likely involve signaling pathways mediated by receptors distinct from those involved in *β*-glucan recognition.

Likewise, even though in all treatments the gene expression of *IL-6*, *IL-13*, *IL-17*, and *TLR-4* was tested but not detected, this might be due to the specific conditions of the experiment or their sensitivity to LPS and FS-PEWS treatment. The lack of significant changes in these cytokines and receptors suggest that the primary inflammatory response in this model might be more localized to IL-8 and NF-kB pathways, findings that align with previous studies using mostly immune cell models demonstrating the interaction of *β*-glucans with *TLR-2* and *mTOR,* but no evidence exists with colonic cell models so far [33,48]. We therefore assume that in our model, LPS activation likely triggered *TLR-2* signaling (since *TLR-4* was not detected), leading to the downstream activation of *NF-kB*, which in turn upregulated pro-inflammatory cytokines such as *IL-1β* and *IL-8*. The significant increase in *mTOR* expression suggests that FS-PEWS may modulate growth and metabolic signaling pathways, potentially through nutrient sensing and immune signaling mechanisms, while at the same time, the significant downregulation of *NF-kB* in FS-PEWS-treated cells compared to LPS-stimulated cells suggests that FS-PEWS might inhibit *NF-kB* activation, thereby reducing inflammation.

Taking into consideration the results obtained from this study and their interpretation, the considerable inter-individual variation observed among the fecal donors has to be taken into account. This variability highlights the intricate and multifactorial nature of immune responses and intestinal barrier function. In addition to the direct role of *β*-glucans, the distinct fermentation patterns observed among the three mushroom-based substrates highlight the unique microbial mechanisms associated with each form. These mechanisms lead to the synthesis of beneficial short-chain fatty acids (SCFAs) through diverse metabolic pathways. The results indicate that the fermentation process of *P. eryngii* substrates—whether as PEWS, PEWSD, or PEWSE—triggers intricate metabolic interactions, with each variant exerting a specific influence on microbial dynamics and the generation of metabolites [18]. In particular, we previously showed that *P. eryngii* fermentation in the elderly can enhance the abundance of beneficial microbes such as *Bifidobacterium* spp. and the butyrate producer *Faecalibacterium prausnitzii*, leading to the increased production of acetate and butyrate, which play crucial roles in maintaining gut barrier integrity and modulating inflammatory responses [18,19]. Particularly, *F. prausnitzii* contributes to the preservation of intestinal barrier integrity by enhancing the synthesis of TJ, expression of *ZO-1*, and proliferation of colonic epithelial cells [49]. Although *β*-glucans are the primary bioactive constituents of *P. eryngii* and are likely central to its observed effects, additional compounds such as chitin, *α*-glucans, and phenolic compounds may also play contributory roles [50]. While our previous metabolomics analysis [18] did not detect metabolites directly associated with chitin or phenolic compounds across the fermentation supernatants, the well-established prebiotic, immunomodulatory, and antioxidant activities of these compounds indicate their potential to exert indirect effects by modulating microbial activity [51,52,53].

Importantly, while all *P. eryngii* substrates used in this study underwent lyophilization to preserve their bioactive content, the observed functional differences in barrier integrity and immunomodulatory activity are unlikely to be attributed to the freeze-drying process itself, but rather to the specific properties of the FSs derived from each substrate. Lyophilization serves to retain polysaccharides, phenolics, and antioxidants, preventing degradation that could arise from alternative drying methods [54,55]. However, since the FSs were the test compounds in this study, their effects on gut barrier function and immune modulation were driven by the metabolic composition of the FSs rather than the preservation method of the original substrates.

While lyophilization was applied uniformly across all *P. eryngii* preparations to maintain consistency, it is worth noting that different drying techniques or the use of fresh, non-lyophilized mushrooms could potentially influence the initial fermentation process. Previous studies have reported that alternative drying methods, such as hot-air drying, may degrade key bioactive components, thereby affecting their microbial utilization and metabolic fate [54,55]. However, given that our study specifically assessed the impact of the FSs on intestinal barrier and immune responses, rather than the fermentation process itself, a direct comparison with fresh or differently processed mushrooms falls beyond its scope. Future studies could explore whether variations in substrate processing impact the metabolite composition of fermentation supernatants and subsequently influence host cellular responses.

The intact food matrix of FS-PEWS offers a more diverse and synergistic bioactive profile than its digested or *β*-glucan-enriched extracted forms. This complexity likely underpins its superior capacity to foster diverse microbial interactions and generate beneficial metabolic outputs, such as the enhanced production of SCFAs. The substrate-specific differences observed in fermentation outcomes further highlight the distinct metabolic contributions of each *P. eryngii* form. Collectively, these findings underscore the value of preserving the whole food matrix, as exemplified by FS-PEWS, in promoting more robust and beneficial microbial interactions compared to its processed counterparts.

## 5. Conclusions

In conclusion, we showed that fecal fermentation supernatants from *P. eryngii* mushrooms, including whole mushroom, digested, and extracted forms, effectively protected intestinal barrier integrity, with the whole mushroom food matrix showing the greatest efficacy. Interestingly, the enhanced expression of *TJ* genes in FS-PEWS-treated Caco-2 cells, particularly in the context of LPS-induced stress, coupled with its balanced modulation of cytokine levels, affirmed its promise as a potent intervention for intestinal inflammation and barrier dysfunction. Overall, the combined treatment of FS-PEWS followed by LPS stress induced a robust immune response characterized by both pro-inflammatory and anti-inflammatory cytokine production and intestinal immune activation, suggesting a complex effect between LPS and *P. eryngii* mushroom components on immune signaling pathways. Given the increasing prevalence of intestinal barrier dysfunction and inflammation in the aging population, these results highlight the potential of FS-PEWS as a therapeutic strategy to enhance gut health and immune function in elderly individuals. Future research should delve into the mechanistic pathways underlying these effects and validate these findings in *in vivo* models.

## Figures and Tables

**Table 1:** Sequences of primers used for quantitative real-time PCR.

Gene	Primer Sequences (5′–3′)	Reference
Human *β-actin* F	GCGCGGCTACAGCTTCA	[20]
Human *β-actin* R	CTTAATGTCACGCACGATTTCC	[20]
Human *ZO-1* F	TTCACGCAGTTACGAGCAAG	[21]
Human *ZO-1* R	TTGGTGTTTGAAGGCAGAGC	[21]
Human *Occludin* F	ACAAGCGGTTTTATCCAGAGTC	[21]
Human *Occludin* R	GTCATCCACAGGCGAAGTTAAT	[21]
Human *Claudin-1* F	TGGTCAGGCTCTCTTCACTG	[21]
Human *Claudin-1* R	TTGGATAGGGCCTTGGTGTT	[21]
Human *IL-1β* F	ACAGATGAAGTGCTCCTTCCA	[22]
Human *IL-1β* R	GTCGGAGATTCGTAGCTGGAT	[22]
Human *TNF-α* F	TCTCGAACCCCGAGTGACAA	[23]
Human *TNF-α* R	TATCTCTCAGCTCCACGCCA	[23]
Human *IFN-γ* F	ATCCAGTTACTGCCGGTTTG	[24]
Human *IFN-γ* R	GAAGCACCAGGCATGAAATC	[24]
Human *IL-6* F	AGACAGCCACTCACCTCTTCAG	NM_000600
Human *IL-6* R	TTCTGCCAGTGCCTCTTTGCTG	NM_000600
Human *IL-8* F	GAGAGTGATTGAGAGTGGACCAC	NM_000584
Human *IL-8* R	CACAACCCTCTGCACCCAGTTT	NM_000584
Human *IL-10* F	GGAGAACCTGAAGACCCTCA	[25]
Human *IL-10* R	GATGTCAAACTCACTCATGGC	[25]
Human *IL-13* F	ACGGTCATTGCTCTCACTTGCC	NM_002188
Human *IL-13* R	CTGTCAGGTTGATGCTCCATACC	NM_002188
Human *IL-17* F	CGGACTGTGATGGTCAACCTGA	NM_002190
Human *IL-17* R	GCACTTTGCCTCCCAGATCACA	NM_002190
Human *Dectin-1* F	AACCACAGCTACCCAAGAAAAC	[26]
Human *Dectin-1* R	GGGCACACTACACAGTTGGTC	[26]
Human TLR-2 F	CTTCACTCAGGAGCAGCAAGCA	NM_003264
Human TLR-2 R	ACACCAGTGCTGTCCTGTGACA	NM_003264
Human TLR-4 F	CCCTGAGGCATTTAGGCAGCTA	NM_138554
Human TLR-4 R	AGGTAGAGAGGTGGCTTAGGCT	NM_138554
Human NF-kB F	GGGGATGGTGAGAAGGTTGG	NM_001319226.2
Human NF-kB R	GCAGTGCCATCTGTGGTTGA	NM_001319226.2
Human CR3 F	AAGTCCTCGTTGTCCGTTCC	MW027613.1
Human CR3 R	CTGCAGCCATTTAACAGCCC	MW027613.1
Human mTOR F	GCCGCGCGAATATTAAAGGA	NM_001386500.1
Human mTOR R	CTGGTTTCCTCATTCCGGCT	NM_001386500.1

## Data Availability

The data presented in this study are available on request from the corresponding author due to ethical restrictions and privacy considerations regarding participant data.

## Supplementary Materials

The following supporting information can be downloaded at: https://www.mdpi.com/article/10.3390/biomedicines13020430/s1, Table S1: Concentration detection levels of cytokines in V-Plex panel (pg/mL), Table S2: The relative expression of *TJ* genes in response to incubation (2% *v*/*v* for 48 h) with FS-NC, FS-PEWS, FS-PEWSD, and FS-PEWSE from a total of five fecal donors in the LPS-stimulated (100 ng/mL for 24 h) Caco-2 cells.
