# Supplementary material for: Effects of In Vitro Fermented Pleurotus eryngii on Intestinal Barrier Integrity and Immunomodulation in a Lipopolysaccharide-Induced Colonic Model
Source: Biomedicines. 2025 Feb 11;13(2):430. doi: 10.3390/biomedicines13020430 (PMC11853518; doi:10.3390/biomedicines13020430)
Supplement: Supplementary file 1 [file biomedicines-13-00430-s001.zip › biomedicines-3459996-supplementary.pdf]

Table S1. Concentration detection levels of cytokines in V-Plex panel (pg/mL)

Analyte	Range of measurement (pg/mL)
IFN-γ	39.9 – 556
IL-1β	11.2 - 225
IL-2	3.19 – 392
IL-6	8.13 – 271
IL-10	3.80 – 134
IL-12p70	5.64 – 173
TNF-α	1.77 – 76.3

Table S2. The relative expression of *TJ* genes in response to incubation (2%v/v for 48 h) with FS-NC, FS-PEWS, FS-PEWSD, and FS-PEWSE from a total of five fecal donors in the LPS-stimulated (100 ng/ mL for 24 h) Caco-2 cells.

	**Untreated cells**	**Cells+LPS**	**FS-NC**	**FS-PEWS**	**FS-PEWSD**	**FS-PEWSE**
ZO-1	1.00	0.85 (0.76,0.96)<br>a	0.92 (0.64,0.98)<br>a	1.20 (0.92,1.41)<br>a, *, ¥	0.88 (0.76,1.03)<br>a, *, †	0.95 (0.72,1.13)<br>†
occludin	1.00	0.87 (0.65,0.96)<br>a	0.95 (0.84, 1.10)<br>*	1.28 (1.12,1.42)<br>a, *, ¥	0.88 (0.71,1.16)<br>*, †	1.04 (0.87,1.10)<br>*, †, ‡
claudin-1	1.00	0.85 (0.65,0.98)<br>a	0.83 (0.71,0.94)<br>a	1.14 (0.97,1.27)<br>a, *, ¥	0.96 (0.86,1.08)<br>*, ¥, †	1.06 (0.88,1.23)<br>*, ¥

Data are expressed as mRNA expression (normalized to that of *β*-actin) relative to untreated cells as the mean ± SD of two independent experiments. Untreated cells: culture cells without any effect, Cells+LPS: culture cells stimulated only with LPS, FS-NC: FSs of the negative control (basal medium with no carbohydrate source); FS-PEWS: *P. eryngii* lyophilized powder from the whole fruit body (mushroom); FS-PEWSD: enzyme-digested PEWS; FS-PEWSE: hot-water extract of PEWS; a: statistically significant compared to untreated cells; * : statistically significant compared to LPS; ¥ : statistically significant compared to FS-NC/LPS; † : statistically significant compared to FS-PEWS/LPS; ‡ : statistically significant compared to FS-PEWSD/LPS; $p<0.05$ (Wilcoxon signed-rank test).
